# Preliminary analysis of chromatin accessibility in osteosarcoma tissues

**DOI:** 10.1016/j.clinsp.2026.101115

**Published:** 2026-09-05

**Authors:** Fan Ye, Po Hu

**Affiliations:** Orthopedics Department, The First Affiliated Hospital of Nanyang Medical College, Henan, China

**Keywords:** Osteosarcoma, Chromatin accessibility, JunB, Apoptosis, Invasion

## Abstract

Osteosarcoma (OS) is highly invasive, metastatic, and lacks effective therapies, leading to poor prognosis. This study aims to dissect the core epigenetic features of OS from the perspective of chromatin accessibility to elucidate its pathogenesis and identify novel therapeutic targets. ATAC-seq was used to map the global chromatin accessibility profiles of OS tissues versus normal controls. We identified OS-specific aberrantly accessible chromatin regions and delineated enriched transcription factor binding motifs. Key transcription factors were validated by Western blotting and flow cytometry. Notably, in regions with enhanced accessibility, the binding probability of AP-1 family members (including Atf3, Fra1, Fra2, JunB, BATF, AP-1, Jun-AP1, Bach2, and CTCF) was significantly elevated. Further analysis indicates that JunB can inhibit apoptosis of OS cells and enhance their invasive ability, suggesting that it may be related to tumor progression. Overall, JunB, as a candidate molecule with potential research value, is worthy of further verification in subsequent in vivo models and clinical samples to determine its feasibility as an intervention target for OS.

## Highlights

• Revealed the global chromatin accessibility landscape of osteosarcoma.
• Identified AP-1 family members, especially JunB, as key regulatory drivers.
• Validated JunB as a promising therapeutic target promoting tumor proliferation and invasion.

## Introduction

OS is a malignant tumor originating from mesenchymal cells and ranks first among primary malignant tumors in children and adolescents.^1^ Although its incidence is low, its highly aggressive biological behaviors ‒ such as rapid proliferation, early hematogenous metastasis, and resistance to conventional chemotherapy ‒ make it one of the most challenging diseases in the field of bone oncology. OS cases are typically resistant to traditional chemotherapeutic drugs, and high-dose chemotherapy leads to severe side effects, including neutropenia, infectious complications, and thrombocytopenia.^2^ Currently, the standard treatment regimen for OS includes extensive surgical resection, neoadjuvant chemotherapy, and adjuvant chemotherapy. Although local control rates are acceptable, the tumor exhibits strong local invasiveness and a high propensity for early lung metastasis, with an overall recurrence rate of 30%–50%. At initial diagnosis, 20%–30% of cases are already metastatic or recurrent, and the prognosis is poor, with approximately 68% of patients with localized disease surviving beyond 5-years.^3^ More critically, over the past four decades, there has been little substantial progress in treatment options for relapsed/refractory OS, underscoring the urgent need to explore novel therapies. The fundamental reason lies in the high heterogeneity of tumor cells and their complex molecular and genetic mechanisms, which make it extremely difficult to devel op a single effective therapeutic approach in the clinical setting.^4^ Therefore, elucidating the metastatic mechanisms of OS is of critical importance for improving its clinical prognosis.

The maintenance of cellular states, differentiation, and disease pathogenesis are all fundamentally driven by changes in gene expression, which are in turn controlled by precise transcriptional regulatory programs.^5^ Transcription factors can regulate gene programs, and by controlling only a few specific key transcription factors in a cell, one can determine cell type specificity and gene expression status.^6^ The AP-1 transcription factor family (including c-Jun, JunB, JunD, cFos, FosB, Fra-1, and Fra-2) can form homodimers or heterodimers to participate in complex transcriptional regulatory networks, thereby responding to various intracellular and extracellular signals.^7^^,^^8^ In recent years, accumulating evidence has indicated that AP-1-mediated transcriptional regulation is closely associated with the development of OS. For example, monocyte chemoattractant protein-1 promotes cancer cell migration through the c-Raf/MAPK/AP-1 pathway and MMP-9 production in OS.^9^ In OS, FOSL1 not only promotes tumor growth but also, upon its silencing, significantly weakens cell proliferation, invasion, and migration by inhibiting the ERK/AP-1 signaling pathway.^10^ Furthermore, glabridin inhibits OS migration and invasion by suppressing p38- and JNK-mediated CREB-AP1 complex formation.^11^ Although these studies have revealed important functions of AP-1 in OS, the specific roles and mechanisms of key AP-1 family members (such as JunB, JunD, FosB, and Fra-2) in OS remain largely unknown and urgently require further exploration. It is noteworthy that the functions of AP-1 family members differ significantly due to variations in dimer composition, expression regulation, DNA binding and transcriptional activity, as well as the intrinsic properties of the proteins themselves.^12^, ^13^, ^14^ Therefore, systematically dissecting the cooperative and differential roles of these key family members in OS is of substantial scientific necessity.

To this end, this study will first employ genome-wide visualization analysis of chromatin accessibility to delineate the global landscape of chromatin accessibility changes in OS tissues. On this basis, we will further identify AP-1 as a key transcription factor driving OS pathogenesis. Finally, combined with in vitro experiments, we will elucidate the specific role and molecular mechanism of its core family member, JunB, in regulating the malignant behaviors of OS cells.

## Materials and methods

### Human OS tissue specimens

In this study, three OS tissue specimens were collected from patients who underwent surgical resection at the Department of Orthopedics, The First Affiliated Hospital of Nanyang Medical College, between January 2022 and December 2025. None of the patients had received chemotherapy or radiotherapy prior to surgery. Meanwhile, three normal bone tissue specimens obtained during surgical procedures for bone trauma or bone tumor resection were collected as controls. Written informed consent was obtained from all patients or their legal guardians prior to specimen collection. The study protocol was approved by the Ethics Review Committee of The First Affiliated Hospital of Nanyang Medical College (Approval n° 2022-xxgnk201).

### U-2OS cell

The human U-2OS osteosarcoma cell line used in this study was purchased from the Cell Bank of the Chinese Academy of Sciences (Catalog n° SCSP-5030). The cells were cultured in McCoy's 5A medium supplemented with 10% fetal bovine serum and routinely passaged in an incubator at 37 °C with 5% CO₂.

### Assay for transposase accessible chromatin with high-throughput sequencing (ATAC-seq)

Three biological replicates of OS and normal tissue samples from each group were used to construct ATAC-seq libraries. Raw reads were filtered using Trimmomatic software with default parameters. Subsequently, the clean data were aligned to the reference genome mm10_gencode using the BWA-MEM algorithm. The resulting BAM file from the alignment analysis was used as the input file, and peak calling was performed using MACS2 software with a filtering threshold of *q* < 0.05. The average read signal over all peaks was calculated using deepTools software. Motif analysis was conducted using the findMotifsGenome.pl tool of HOMER. Peaks were annotated by bedtools.^15^ Finally, differential accessible peak was assessed using DESeq2. Region was called differentially accessible if the absolute value of the log2 fold change was 1 at an *p* < 0.05.

### Small interfering RNA transfection

Logarithmic U-2OS cells were seeded into 6-well plates at 2.5 × 10^5^ cells/well. After 24hours (60%–80% confluence), medium was replaced with serum-free and antibiotic-free MEM. Following 6-hours of starvation, medium was changed to serum/antibiotic-free MEM. For siRNA transfection, 250 μL of serum/antibiotic-free MEM diluted siRNA or negative control to 50 nmoL/L in tubes A and incubated for 5 min. Meanwhile, tubes B received 250 μL MEM and 5 μL iMAX, mixed, and incubated for 5-min. Contents of A and B were combined, incubated for 15min, added to wells. After 24hours, cells were harvested. The sequences are as follows: sense strand, CGACUACAAACUCCUGAAATT; antisense strand, UUUCAGGAGUUUGUAGUCGTT. We thank the reviewer again for this valuable comment.

### Overexpression plasmid

The U-2OS cells in the logarithmic growth phase were seeded at a density of 2.5 × 10^5^ cells per well into a 6-well plate. After 24-hours (with a density of 60%–80%), the medium was replaced with serum-free medium. After 6-hours of starvation, the medium was changed to serum-free and antibiotic-free MEM. During transfection, 125 μL Opti-MEM, 2.5 μg plasmid and 5 μL P3000 enhancer were added to tube A, and 125 μL Opti-MEM and 5 μL Lipofectamine 3000 were added to tube B. They were mixed well and then combined. After incubation at room temperature for 15minutes, each well was added drop by drop. The cells were harvested 24-hours later for subsequent experiments.

### Hematoxylin and eosin staining (HE)

Normal bone and OS tissues were fixed in 4% paraformaldehyde for 24hours, then paraffin-embedded and cut into 4 μm sections. After deparaffinization, sections underwent two staining procedures. Conventional staining: hematoxylin for 5-minutes, differentiation in 1% hydrochloric acid alcohol for 30 seconds, ammonia water rinse, and counterstaining with 0.5% eosin for 2 minutes, then dehydrated and mounted with neutral gum. Special staining: Weigert's hematoxylin for 8-minutes, Ponceau-fuchsin for 5-minutes, differentiation with 1% phosphomolybdic acid for 2minutes, counterstaining with aniline blue for 5minutes, and mounted with neutral balsam. Histopathological morphology was observed using an advanced microscope.

### Western blotting

Total protein was extracted from U-2OS cells using Radioimmunoprecipitation Assay (RIPA) buffer containing Phenylmethanesulfonyl Fluoride (PMSF) (both from Boster Biological Technology, China), according to the manufacturer's protocol. Protein concentration was determined using a BCA protein assay kit (Boster Biological Technology, China). Subsequently, proteins were separated by sodium dodecyl sulfate-polyacrylamide gel electrophoresis (SDS-PAGE) and transferred onto PVDF membranes. The membranes were incubated overnight at 4 °C with anti-JunB antibody (1:1000, Proteintech, China) and anti-GAPDH antibody (1:10,000, Proteintech, China), followed by incubation with corresponding secondary antibodies for 1hour at room temperature. Finally, protein signals were detected using a chemiluminescence imaging system (e-BLOT Touch Imager, China).

### Flow cytometry

Remove the supernatant of U-2OS cell culture and transfer it to a centrifuge tube. Wash the adherent cells once with PBS and then aspirate and discard. Add trypsin without EDTA to digest the cells until they become round. Immediately add the original culture supernatant to neutralize the trypsin. Mix by pipetting and transfer all the cells to the same centrifuge tube. Centrifuge at 1000 g for 5-minutes, discard the supernatant, resuspend with PBS and wash once again. Centrifuge again and discard the supernatant. Resuspend with PBS for counting. Take 5 × 10^4^ to 1 × 10^5^ cells. Centrifuge and discard the supernatant. Add 195 μL binding solution to resuspend, then add 5 μL Annexin V-FITC (Beyotime, C1062S) and 10 μL PI. Mix well and incubate at room temperature in the dark for 10‒15 minutes. After incubation, perform the assay within 1-hour.

### Cell invasion assay

Transwell chambers (8 μm pore size) were coated with Matrigel. U-2OS cells resuspended in serum-free medium (5 × 10^4^ cells/well) were added to the upper chamber, while medium containing 10% FBS was placed in the lower chamber as a chemoattractant. After transfection of siR-JunB/OV-JunB into U-2OS cells and incubation for 24-hours, the cells were fixed with 4% paraformaldehyde and stained with 0.1% crystal violet. The non-invading cells on the upper surface were removed with a cotton swab, and the invading cells on the lower surface were counted under a microscope.

### Statistical analysis

ATAC-seq data were analyzed using DESeq2 (v1.38.3) with Benjamini-Hochberg correction for multiple testing. Differentially accessible regions were defined at FDR < 0.05 and |log₂FC| ≥ 1. Peak calling used MACS2 (v2.2.7.1) at *q* < 0.05, and motif enrichment in HOMER was considered significant at FDR < 0.05. For other experiments, statistical significance was determined using two-tailed Student's *t*-test or one-way ANOVA, with *p* < 0.05 considered significant. All data are shown as mean ± SD from at least three independent experiments.

## Results

### Histopathological examination of OS tissues

**Fig. 1:**
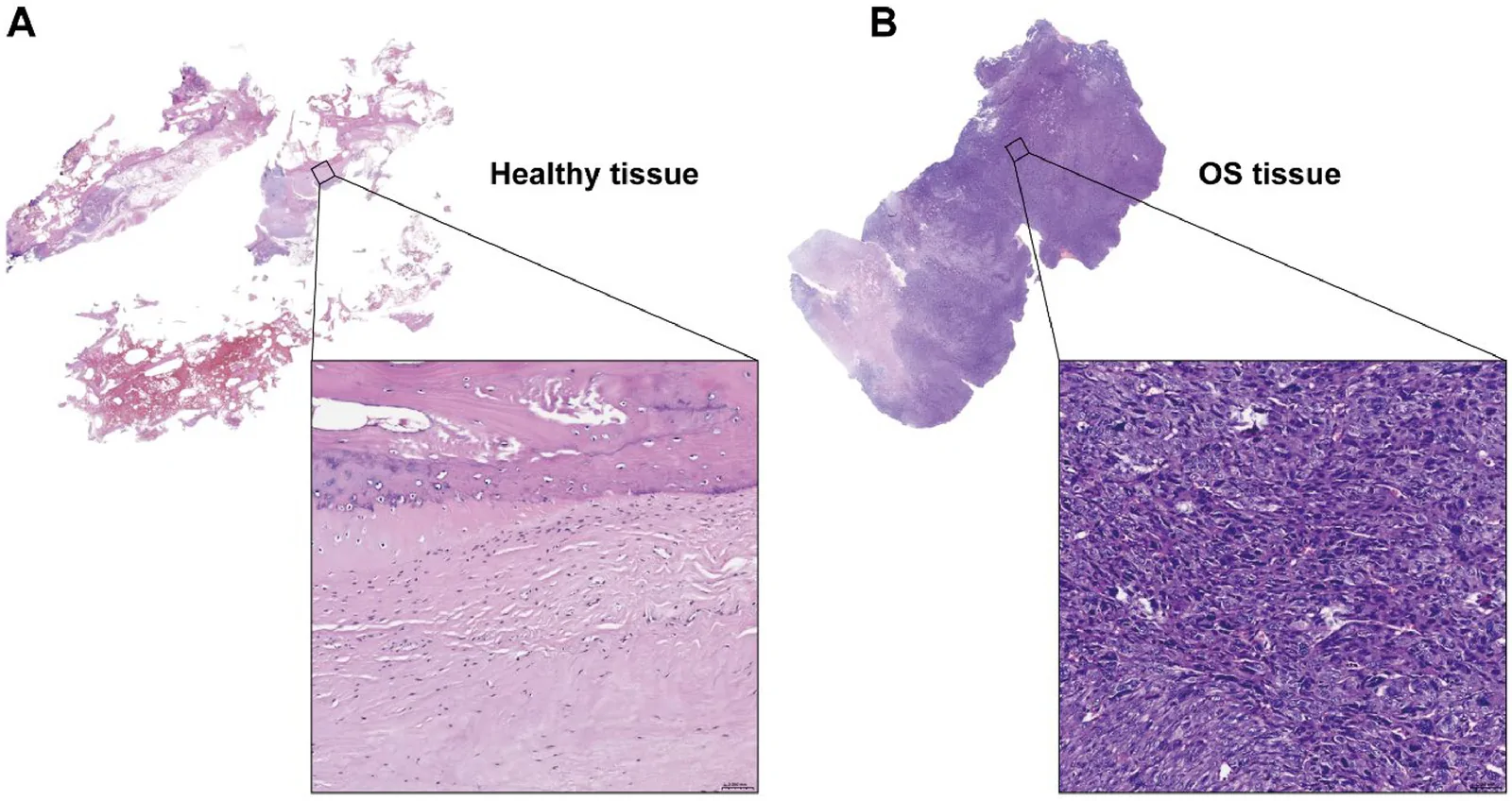
HE-staining of normal bone tissue and OS bone tissue samples. (A) Normal bone tissue. (B) OS tissue.

HEstaining was performed on bone tissue samples obtained from normal controls and OS patients. Microscopic examination revealed that normal bone tissue exhibited regular morphology of osteocytes without obvious abnormalities (Fig. 1A). OS tissue displayed typical histological features of osteosarcoma: the tissue showed a diffuse sheet-like distribution, with significant tumor cell atypia. The tumor cells were predominantly spindle-shaped or irregular in shape, with hyperchromatic nuclei, an increased nuclear-to-cytoplasmic ratio, and numerous mitotic figures (Fig. 1B).

### Chromatin accessibility landscape of OS tissues

**Fig. 2:**
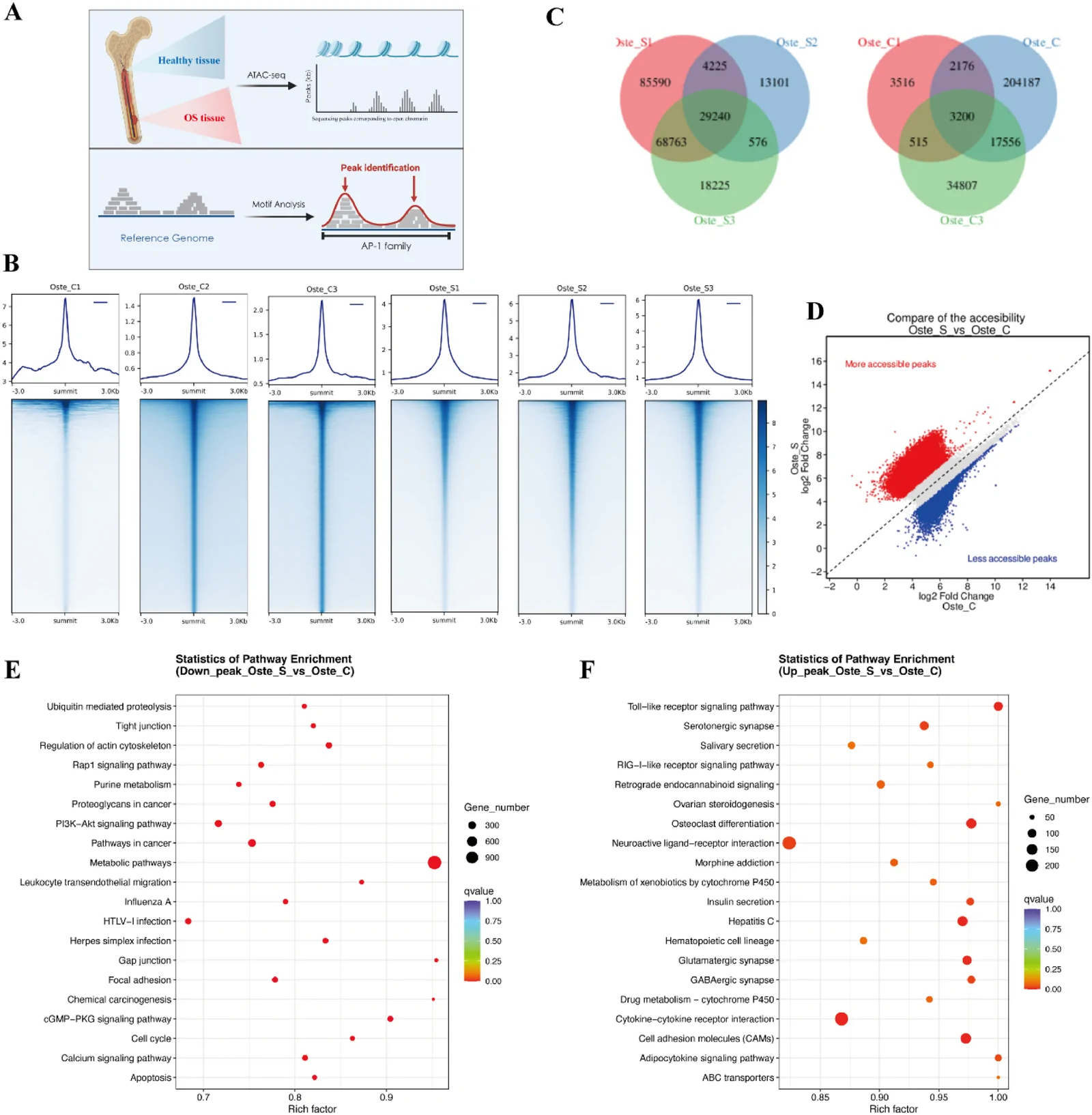
Chromatin accessibility features of OS tissues. (A) Schematic diagram of the ATAC-seq workflow. (B) Venn diagram showing overlapping peaks between normal and OS tissue samples (intersection analysis). (C) Enrichment of ATAC-seq signals at transcription start sites in normal and OS tissues. (D) Volcano plot comparing chromatin accessibility peaks between normal and OS tissues. Red dots represent regions with increased accessibility, and blue dots represent regions with decreased accessibility. (E) KEGG enrichment analysis of genes adjacent to the top 20 differential peaks between normal and OS tissues.

Given that chromatin remodeling is a key mechanism driving large-scale changes in gene expression during bone tumorigenesis, we further analyzed genome-wide chromatin accessibility changes in OS tissues using ATAC-seq, a technique that enables high-throughput quantitative detection of open chromatin regions (Fig. 2B). To evaluate the enrichment quality of peak calling, we first calculated the average signal distribution within normalized peak regions for each sample using deepTools and plotted the corresponding curves. The results showed that reads aligned to the reference genome were highly concentrated in the central regions of the peaks, indicating good enrichment specificity of the peak calling results (Fig. 2C). Subsequently, we identified intersecting peaks across samples. A total of 29,240 common peaks were identified in the three normal tissue samples, whereas only 3200 common peaks were identified in the three OS tissue samples (Fig. 2D). Using DESeq2 to analyze ATAC-seq signals between OS and normal tissues, with a threshold of |log2^FC^| > 1 and *p* < 0.05, we identified thousands of loci with altered chromatin accessibility, including regions with increased and decreased accessibility (Fig. 2D). To explore their functions, the top 20 differentially accessible peaks were annotated using the KEGG database. The results showed that these genes were mainly enriched in signaling pathways such as metabolic pathways, cytokine-cytokine receptor interaction, cell adhesion molecules, and osteoclast differentiation (Fig. 2E‒F). These pathways play critical roles in osteoclast activation and bone resorption, immune cell recruitment in the inflammatory microenvironment, and tumor-bone matrix adhesion and interaction.

### Motif analysis indicated that JunB may be a key transcription factor influencing the pathogenesis of OS

**Fig. 3:**
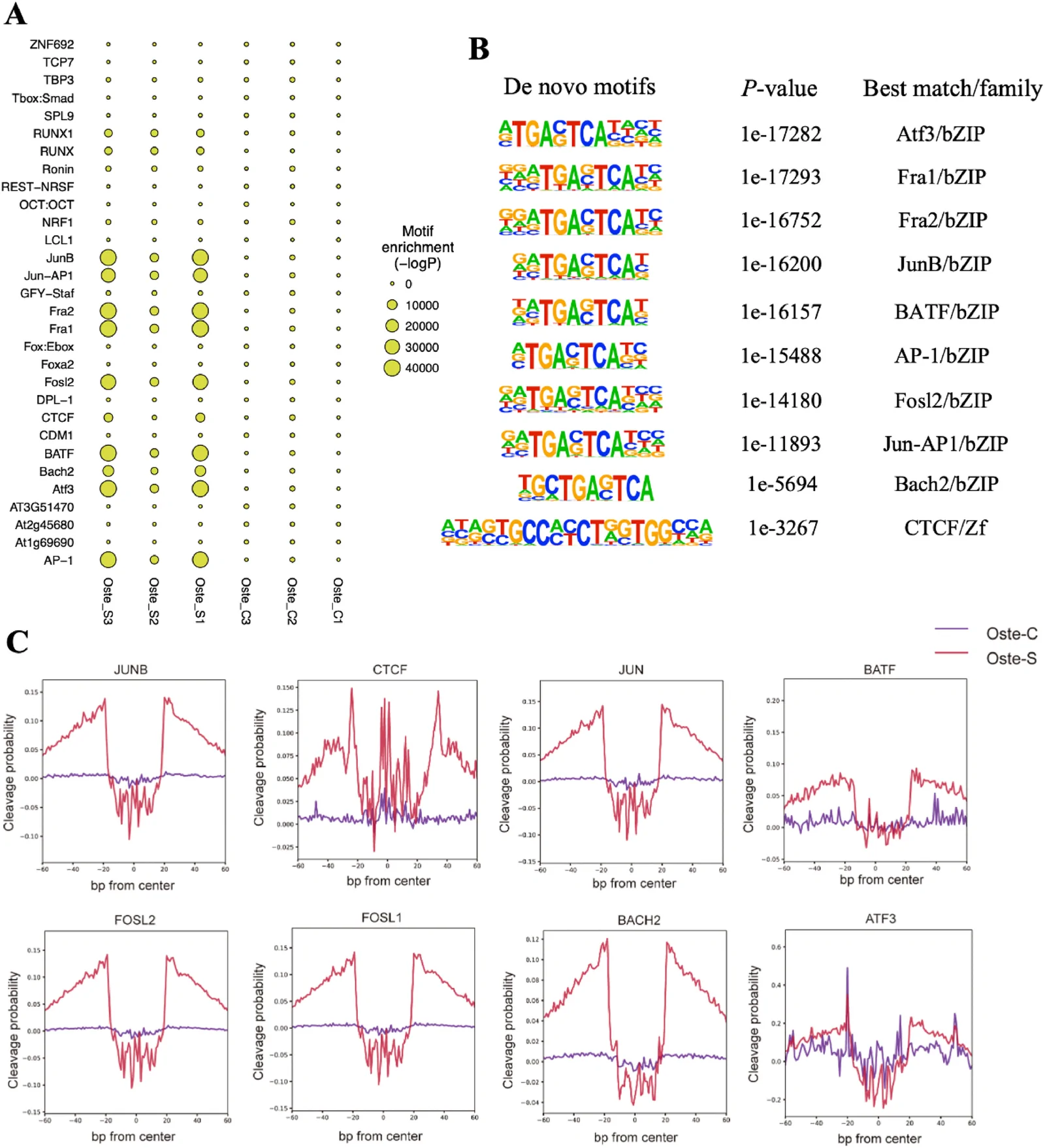
Motif analysis and transcription factor identification in OS tissues. (A‒B) Motif analysis of upregulated peak sequences: showing the motif sequences of AP-1 family members. (C) ATAC-seq-based transcription factor footprint analysis: distribution probability of AP-1 family member binding motifs near the peak summits of Differentially Accessible Regions (DARs).

Chromatin accessibility is closely associated with transcription factor binding and plays a critical role in gene activation and repression. Transcription factors typically bind near gene transcription start sites to regulate the expression of upstream and downstream genes. First, based on *p*-values, the top 20 differentially accessible peak sequences were selected for motif analysis using the findMotifsGenome.pl tool in HOMER software to locate the positions of these motifs on the peaks and identify the corresponding transcription factor binding sites. By comparing the transcription factor motifs enriched in regions with increased chromatin accessibility between normal bone tissues and OS tissues, we found that AP-1 family members accounted for the highest proportion among the top 10 transcription factors (Fig. 3A‒B). ATAC-seq footprint analysis reveals the dynamics of transcription factor binding during genome activation and predicts their precise binding positions at specific sites. Footprint analysis of the genome-wide binding status of the transcription factor AP-1 before and after OS development showed that AP-1 family members (Atf3, Fra1, Fra2, JunB, BATF, AP-1, Jun-AP1, Bach2, and CTCF) exhibited higher binding probability near the peak summits of hyperaccessible regions (Fig. 3C). These results suggest that the AP-1 family may play an important regulatory role in OS development. We reviewed the literature and found that among the other AP-1 family members significantly enriched in our ATAC-seq motif analysis, Fra1^16^ and ATF3^17^ have been relatively well studied in osteosarcoma in terms of their functions and mechanisms. In contrast, both JunB and BATF remain poorly characterized in osteosarcoma. To prioritize among these candidates, we further examined the results of transcription factor footprinting analysis, which revealed that the chromatin accessibility signal at the JunB binding motif was significantly stronger than that at BATF, suggesting that JunB may possess a higher actual binding potential within the open chromatin landscape of osteosarcoma. Based on the combination of the stronger footprinting signal observed in our own data, the lack of osteosarcoma-related functional studies for BATF in the literature, and the preliminary transcriptomic indications linking JunB to cell proliferation and invasion, we selected JunB as the priority candidate for further validation, with the aim of filling the knowledge gap regarding its role in osteosarcoma.

### JunB can promote the progression of osteosarcoma by inhibiting apoptosis in U-2OS cells and enhancing their invasive ability

**Fig. 4:**
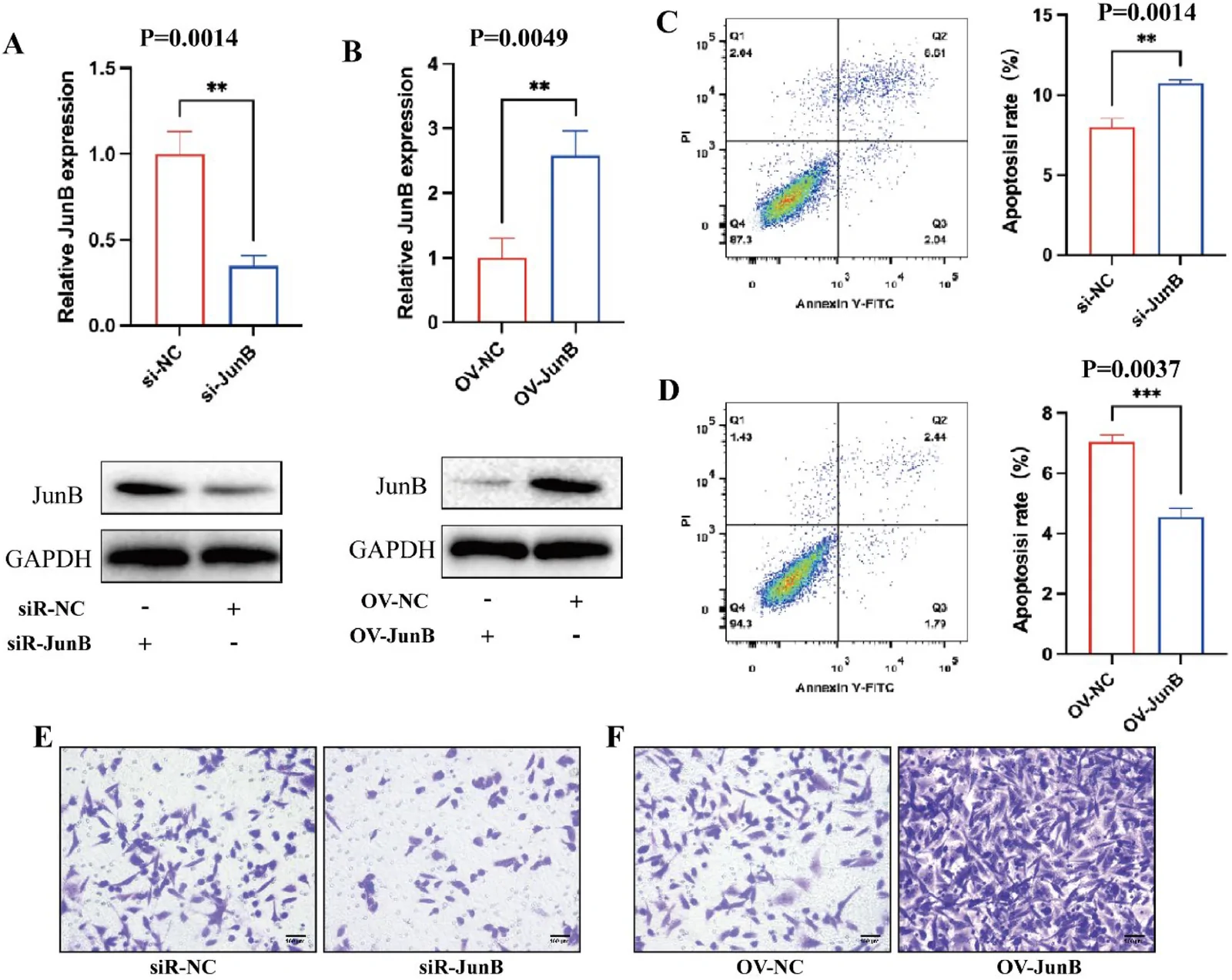
JunB inhibits apoptosis in U-2OS cells and promotes their invasion, thereby facilitating the progression of OS. (A‒B) Protein expression levels following JunB knockdown or overexpression. (C‒D) Effects of JunB knockdown or overexpression on apoptosis of U-2OS cells. (E‒F) Effects of JunB knockdown or overexpression on invasion of U-2OS cells. All data are expressed as mean ± SD from three independent biological replicates (n = 3) *p < 0.05; **p < 0.01; ***p < 0.001.

The U-2OS cell line was further used to investigate the effects of JunB on tumor cell apoptosis and invasion. Western blot analysis showed that JunB protein expression levels were significantly reduced following JunB silencing in U-2OS cells; conversely, JunB protein expression levels were significantly increased upon JunB overexpression, confirming the successful establishment of JunB knockdown and overexpression models (Fig. 4A‒B). Flow cytometry analysis revealed that JunB knockdown significantly induced apoptosis in U-2OS cells, as evidenced by a marked increase in the apoptosis rate (Fig. 4C‒D). Furthermore, Transwell invasion assay results demonstrated that JunB upregulation significantly enhanced the invasive capacity of U-2OS cells, as shown by a significant increase in the number of invading cells (Fig. 4E‒F).

## Discussion

OS is a primary malignant bone tumor that frequently occurs in children and adolescents, characterized by high genomic instability and invasiveness.^18^^,^^19^ Although the combination of surgery and chemotherapy has significantly improved patient outcomes, the five-year survival rate for metastatic and recurrent OS remains below 30%, underscoring the urgent need to explore novel pathogenic mechanisms.^20^ Unlike traditional gene mutation-driven models, the role of epigenetic dysregulation in tumor initiation and progression is increasingly recognized.^21^ Chromatin accessibility, as a key dimension of epigenetic regulation, determines whether transcription factors can bind to DNA, thereby activating or silencing specific gene expression programs.^22^ Studies have shown that dysregulation of chromatin remodeling complexes (such as the SWI/SNF and Polycomb families) is common in mesenchymal lineage tumors associated with skeletal development, and the discovery of histone mutations (oncohistones) in OS provides direct evidence for an “epigenetically driven” tumorigenesis model.^18^ In recent years, single-cell and multi-omics technologies have revealed that OS exhibits distinct cell states driven by different transcription factor networks, and these states are closely associated with patient prognosis and treatment response.^23^^,^^24^ Therefore, systematically dissecting the epigenomic landscape of OS from the perspective of chromatin accessibility is of great value for identifying core regulatory factors and discovering potential therapeutic targets.

ATAC-seq is a high-throughput sequencing technology that analyzes genome-wide chromatin accessibility using the Tn5 transposase, enabling the identification of transcription factor binding regions and nucleosome positions. Focusing on the epigenetic characteristics of OS, this study employed ATAC-seq to systematically map the global chromatin accessibility landscape of OS tissues compared with normal controls. The analysis revealed a substantial number of specific, aberrantly accessible chromatin regions in OS. Through in-depth dissection of transcription factor binding motifs enriched within these aberrantly accessible regions, we successfully identified a series of key transcription factors that may play core regulatory roles during OS pathogenesis. Among the top ten transcription factors, AP-1 family members accounted for the highest proportion, highlighting the central role of this family in reshaping the chromatin landscape of OS. Notably, the binding motif of JunB, an important member of the AP-1 family, was highly enriched in aberrantly accessible regions, suggesting a potential regulatory role for JunB in modulating OS chromatin accessibility and downstream oncogenic gene expression networks. These findings provide correlative epigenetic evidence supporting our previous research and position JunB as a candidate molecular hub linking aberrant chromatin states to malignant phenotypes in OS. Importantly, because ATAC-seq identifies open chromatin and enriched motifs but does not directly demonstrate transcription factor occupancy, we refer to JunB as a putative regulator throughout this study. Future work will focus on experimentally validating JunB occupancy at specific accessible regions via ChIP-seq or CUT&RUN and dissecting the molecular mechanisms by which this regulatory activity drives OS metastasis.

As an important component of the transcription factor complex AP-1, JunB is widely expressed in cells and plays a critical role in regulating various physiological functions and disease-related processes. In terms of cell cycle regulation, JunB promotes cell cycle progression by inducing the expression of cyclin E1 and inhibiting the transcription of the transforming growth factor TGF-β2 gene. Further studies have revealed that high levels of JunB can shift the cellular response to TGF-β2 stimulation from an anti-proliferative to a pro-invasive state, and induce endogenous TGF-β2 production by promoting the translation of TGF-β2 mRNA, thereby enhancing tumor growth and metastasis in mice.^25^ In addition, related studies have shown that transfection with JunB short hairpin RNA (shRNA) inhibits Hepatocyte Growth Factor (HGF)-induced upregulation of MMP-9 and attenuates HGF-mediated cell proliferation and invasion.^26^ The Tumor Microenvironment (TME) plays a critical role in regulating immune responses and influencing the efficacy of immunotherapy. Recent studies have revealed the intricate crosstalk between immune cells and tumor cells within the TME: efferocytosis by macrophages impairs antitumor immunity through the TP63–RAC2 pathway;^27^ SATB2 promotes malignant behavior in pancreatic cancer while suppressing T-cell cytotoxic function;^28^ aberrant activation of the JAK/STAT signaling pathway can remodel the TME to induce immunosuppression and therapeutic resistance, and combining JAK inhibitors with PD-1 blockade holds promise for overcoming such resistance.^29^ Furthermore, TRIM29 and WDR54 promote malignancy in glioblastoma and hepatocellular carcinoma, respectively, through activation of the PI3K/AKT and NF-κB signaling pathways.^30^^,^^31^ Collectively, these findings highlight that a deeper understanding of the interactions among various TME components is of great importance for optimizing immunotherapeutic strategies and improving patient outcomes. Collectively, these studies underscore the critical role of TME components in modulating tumor progression and therapeutic responses. Within this context, our findings further extend this understanding by identifying JunB as a key regulator of osteosarcoma cell behavior. In our experiments, knockdown of JunB expression significantly induced apoptosis in U-2OS cells, as evidenced by a marked increase in the apoptosis rate; conversely, upregulation of JunB expression significantly enhanced the invasive capacity of U-2OS cells, as shown by a significant increase in the number of invading cells. Thus, JunB may serve as a potential and important target for tumor intervention by influencing cell apoptosis and invasion.

However, it should be noted that the above findings are limited by the small sample size and should be considered only as preliminary observations. The limited number of clinical samples restricts the statistical power and generalizability of our conclusions, particularly given the well-recognized inter-individual heterogeneity in chromatin accessibility profiles. Moreover, it is important to emphasize that ATAC-seq reveals chromatin openness as a measure of regulatory potential rather than direct functional regulation. Enriched transcription factor binding motifs, including those for JunB, indicate possible occupancy but cannot replace direct binding assays such as ChIP-seq or reporter gene experiments to establish causal regulatory relationships. Therefore, the conclusions drawn from this study are hypothesis-generating in nature and require systematic validation through large-scale cohort studies, independent datasets, and direct functional assays in the future. By clearly acknowledging these limitations, we hope to assist readers in more objectively assessing the strength and scope of our findings.

## Conclusion

In regions with enhanced chromatin accessibility, the binding probability of AP-1 family transcription factors, including Atf3, Fra1, Fra2, JunB, BATF, AP-1, Jun-AP1, Bach2, and CTCF, was significantly elevated. Further analysis suggests that JunB in this family member may be involved in regulating the apoptosis and invasion abilities of osteosarcoma cells, thereby potentially playing a role in the progression of osteosarcoma.

## CRediT authorship contribution statement

**Fan Ye:** Writing – original draft, Methodology, Data curation. **Po Hu:** Supervision, Conceptualization.

## Declaration of competing interest

The authors declare no conflicts of interest or competing financial/personal relationships related to this work.

## Data Availability

Data will be made available on request.
